# Hologenomic rewiring facilitates dietary adaptation to chitin-rich marine resources in the crab-eating frog

**DOI:** 10.1186/s12983-026-00626-1

**Published:** 2026-07-21

**Authors:** Yi Zhang, Zhi-Heng Chang, Shuang Gan, Shi-Han Wang, Jin-Xia Luo, Long Jin, Xiao-Fei Zhai, Yan-Bo Sun

**Affiliations:** 1 State Key Laboratory of Vegetation Structure, Functions and Construction, Yunnan Key Laboratory of Biological Adaptation, Conservation and Utilization, School of Ecology and Environmental Science, Yunnan University, Kunming, 650500 China; 2 Southwest United Graduate School, Kunming, 650092 China; 3 Key Laboratory of Southwest China Wildlife Resources Conservation (Ministry of Education), China West Normal University, Nanchong, P. R. China; 4 Ministry of Education Key Laboratory for Ecology of Tropical Islands, Key Laboratory of Tropical Animal and Plant Ecology of Hainan Province, College of Life Sciences, Hainan Normal University, Haikou, China

**Keywords:** Host-microbe interactions, Dietary specialization, Chitin digestion, Marine amphibian, Metagenomics, Holobiont

## Abstract

**Background:**

Secondary adaptation of amphibians to marine environments is exceptionally rare. The crab-eating frog, *Fejervarya cancrivora*, is the only known amphibian capable of completing its life cycle in intertidal zones, where it faces dual challenges: high salinity stress and a diet rich in chitinous crab exoskeletons. While osmoregulatory adaptations have been well documented, the synergistic roles of the host’s digestive system and its gut microbiota in this dietary specialization remain unclear.

**Results:**

Here, we integrated histological analysis, comparative transcriptomics, chitinase activity assays, and gut metagenomics to compare *F. cancrivora* with its freshwater congener, *F. multistriata*. We found that *F. cancrivora* has evolved a thicker gastric muscularis and longer gastric villi, consistent with enhanced processing of hard prey. Comparative transcriptomic analysis revealed an expanded repertoire of putative chitinase encoding transcripts (15 vs. 8 non-redundant transcripts), and both gastric and intestinal tissues exhibit significantly higher and more pH-tolerant chitinase activity. In contrast, the gut microbiota of *F. cancrivora* is not enriched for microbial chitin degradation genes, but instead is functionally specialized for lipid metabolism and DNA repair pathways. A controlled feeding experiment confirmed that the microbial enrichment in lipid metabolism is diet-driven, while the DNA repair pathways is largely independent of diet and likely reflects microbiome-intrinsic adaptation to chronic saline stress.

**Conclusions:**

Together, these findings suggest a partially partitioned host-microbiome strategy in which host manages chitin breakdown, while the microbiota optimizes energy harvest and intrinsic stress tolerance. Our findings provide a new paradigm for amphibian marine adaptation, and highlights host-microbiome functional differentiation during niche expansion.

**Clinical trial number:**

Not applicable.

**Supplementary Information:**

The online version contains supplementary material available at https://doi.org/10.1186/s12983-026-00626-1.

## Introduction

The colonization of terrestrial habitats by amphibians represents one of the most pivotal transitions in vertebrate evolution, fundamentally reshaping ecosystems and enabling the diversification of tetrapods [1]. However, despite their ancient evolutionary history spanning nearly 400 million years, secondary adaptation to marine environments from freshwater origins is exceptionally rare among amphibians [2]. The crab-eating frog, *Fejervarya cancrivora*, stands as a remarkable exception, being the only known extant amphibian capable of completing its entire life cycle within intertidal high-salinity environments (up to 30 ppt) [3, 4]. This unique niche presents two interconnected physiological challenges: osmotic stress from salinity and the digestive demands of a specialized diet, where crustaceans, primarily crabs, constitute over 60% of its intake [4, 5]. Unlike the soft-bodied invertebrates (e.g., insects) preyed upon by most amphibians [6, 7], crab exoskeletons are rich in chitin, a recalcitrant polymer that poses significant mechanical and enzymatic hurdles for digestion [8]. This dual pressure makes *F. cancrivora* an ideal model to investigate how host organisms and their associated microbial communities co-evolve to overcome extreme environmental and dietary challenges [9–11].

Previous research on *F. cancrivora* has primarily focused on its osmoregulatory adaptations, such as specialized skin ion transport and renal function [12, 13]. However, the integrated roles of host digestive morphology, endogenous enzyme activity, and gut microbiome in enabling its crab-specialized diet remain largely uncharacterized. For example, while the expansion of chitinase genes is known to facilitate dietary adaptation in some mammals [14], the expression, activity, and functional significance of these enzymes in the digestive tract of *F. cancrivora*, particularly under high-salinity conditions, have not been systematically investigated. Similarly, the contribution of its gut microbiota to chitin degradation, nutrient metabolism, or stress tolerance is completely unknown.

In recent years, host-microbe co-adaptation is increasingly recognized as a key strategy for vertebrates facing novel environmental pressures [15, 16]. The gut microbiota provides a flexible metabolic pool that can rapidly evolve, allowing hosts to expand their dietary niche and adapt to extreme conditions, as seen in bamboo-feeding pandas [17] or snail-specialized snakes [16]. However, systematic studies of such co-adaptation in the context of amphibian marine invasion are lacking.

Here, we address this gap by integrating histological analysis, comparative transcriptomics, enzyme activity assays, and gut metagenomics to systematically compare *F. cancrivora* with its closely related, freshwater-dwelling congener, *F. multistriata*. We hypothesize that the successful colonization of the marine intertidal niche by *F. cancrivora* is driven by coordinated host-microbe functional partitioning. Specifically, we predict that (1) the host has evolved morphological and molecular adaptations, such as a reinforced digestive tract and an enhanced, pH-tolerant chitinase system, to mechanically and chemically process chitin-rich prey; and (2) the gut microbiota has concurrently specialized to provide complementary functions, optimizing nutrient (especially lipid) extraction and enhancing resilience to the genotoxic stress of a high-salinity environment. By testing this model, this study aims to elucidate the molecular and ecological basis of amphibian marine adaptation and provide novel insights into the role of host-microbe co-evolution in vertebrate niche expansion.

## Materials and methods

### Sample collection

Adult individuals of *F. cancrivora* (FC; *n* = 30) were collected from intertidal mangrove zones in Zhanjiang, Guangdong (21.5536°N, 109.7636°E; *n* = 20; dry and wet seasons) and Haikou, Hainan (19.9517°N, 110.5761°E; *n* = 10; dry season). For comparison, adult *F. multistriata* (FM; *n* = 10) were collected from freshwater paddy fields in Nanchong, Sichuan (30.8008°N, 106.0472°E; wet season). All procedures were approved by the Animal Ethics Committee of Yunnan University (Approval No. YNU20230645). Following humane double-pithing, stomach and intestinal tissues were excised. Segments were either fixed in 10% neutral-buffered formalin for histology, or snap-frozen in liquid nitrogen for transcriptomics and enzyme assays. Gut contents were collected aseptically and snap-frozen for metagenomics.

### Histological analysis

Fixed tissues were dehydrated, paraffin-embedded, sectioned at 4 μm, and stained with hematoxylin and eosin (H&E) following standard protocols [18]. Stained sections were digitized using an Olympus VS200 slide scanner. Morphological parameters, such as gastric muscle thickness (measured at three equidistant points), gut villus height (from crypt base to villus tip), and epithelial cell height (from basement membrane to apical surface), were quantified using OlyVIA 3.4.1 software (Olympus) [19]. To ensure statistical robustness, measurements were performed blindly by two independent observers across five non-overlapping fields per sample (*n =* 3).

### Transcriptome sequencing and analysis

Total RNA was extracted from stomach and gut tissues (*n* = 5 per species per tissue) using TRNzol Reagent (Tiangen). cDNA libraries were prepared and sequenced on an Illumina NovaSeq 6000 platform (PE150). Raw reads were processed with Trimmomatic v0.40 [20]. Clean reads were *de novo* assembled using Trinity v2.14.0 [21], and completeness and quality were evaluated using BUSCO v5.8.2 [22] against the tetrapoda_odb10 database (completeness > 80% required). Bowtie2 v2.5.4 [23] was employed to map the clean reads back to the assembled transcripts to evaluate the representativeness of the splicing results. Redundant transcripts were clustered using CD-HIT v4.8.1 with a sequence similarity threshold of 95% [24], generating a non-redundant unigene set. Open Reading Frames (ORFs) were predicted using TransDecoder v5.7.1 and annotated against the NR, Swiss-Prot, and KEGG databases (E-value < 1e-5) using the Trinotate pipeline v4.0.2 [25, 26]. Orthologous relationships between FC and FM were identified using a reciprocal best hit (RBH) approach with BLASTP [27]. Differential expression analysis was conducted using DESeq2 v1.34.0 [28], with genes considered significantly differentially expressed at padj < 0.05 and |log2(fold change)| > 1. GO enrichment analysis was performed using clusterProfiler v4.10.1 [29].

To identify putative chitinase encoding transcripts, an orthology analysis was performed using OrthoFinder v3.1.0 [30], integrating our transcriptome-derived proteins with reference genomes of FM, *Quasipaa spinosa* (QS), and *Xenopus tropicalis* (XT). Orthogroups containing known vertebrate chitinase domains were identified, and candidate chitinase encoding transcripts were extracted, with redundant isoforms removed based on sequence similarity (> 95% identity via CD-HIT). A maximum likelihood phylogenetic tree was constructed using IQ-TREE2 [31] to resolve evolutionary relationships among these sequences, with the optimal substitution model (LG + G4) selected via ModelFinder and branch support assessed using 1000 ultrafast bootstrap replicates.

### Chitinase activity assay

Chitinase activity in stomach and gut tissue homogenates (*n* = 5 per species) was measured using a Chitinase Assay Kit (Solarbio) with colloidal chitin as the substrate. To assess pH tolerance, reactions were conducted at pH 3.0, 4.0, and 5.0 for stomach tissues, and pH 4.0, 5.0, and 6.0 for gut tissues. Activity was normalized to total protein concentration (BCA assay) and expressed as U/mg protein, where 1 U = 1 µmol of N-acetylglucosamine (GlcNAc) released per minute. All assays were performed with three technical replicates.

### Metagenomic sequencing and analysis

Genomic DNA was extracted from gut contents of 10 individuals per sampling group. Following DNA quality control and sequencing, a total of 46 viable metagenomic samples were retained for downstream analysis (FC_G_W = 10, FC_G_S = 10, FC_H_W = 10, FM = 9, FC_in_lab = 7). DNA isolation was performed using the FastPure Feces DNA Isolation Kit with an additional bead-beating step (30 s at 60 Hz). Libraries were prepared and sequenced on Illumina NovaSeq Xplus platform (PE150), generating ~ 10 Gb raw data per sample. Reads were processed with fastp v0.23.0 [32] and mapped to host transcriptomes using BWA v0.7.17 [33] to remove host contamination.

Clean microbial reads were co-assembled per species using MEGAHIT v1.1.2 [34]. The optimal k-mer size was determined by evaluating assembly completeness using QUAST v5.2.0 [35]. ORFs were predicted using Prodigal v2.6.3 [36] in metagenome mode, and a non-redundant gene set was constructed with CD-HIT (90% identity) [24]. Gene abundances (RPKM) were calculated by mapping reads back to the gene catalog using SOAPaligner v2.21 [37]. Taxonomic assignment was performed against the NCBI NR database, and functional annotations were made using the CAZy v12 [38] and KEGG database v94.2 [38].

### Controlled feeding (transplant) experiment

To disentangle the effects of diet and environment on the gut microbiome, a subset of wild-caught FC (*n* = 7) from the Zhanjiang population was transferred to the laboratory. These frogs were housed in aquaria with seawater from their native habitat and fed a diet of crickets (*Gryllus sp.*) for four weeks. Following this acclimation period, their gut contents were collected and subjected to metagenomic sequencing and KEGG pathway enrichment analysis as described above. The microbiomes of these lab-fed frogs were compared to those of wild FC from the same original population.

### Statistical analysis

All statistical analyses were performed in R v4.2.2. Alpha diversity indices (Chao1, Shannon-Wiener) were calculated using vegan package v2.6-4 (https://github.com/vegandevs/vegan), with differences between groups compared using the Wilcoxon rank-sum test. Beta diversity (Bray-Curtis dissimilarity) was visualized via principal component analysis (PCoA) and tested using ANOSIM (999 permutations). Differential abundance of microbial genera and KEGG pathways was assessed using the Wilcoxon rank-sum test. Correlations between host gene expression (from RNA-seq) and microbial pathway abundance were evaluated using Pearson’s correlation coefficient. P-values < 0.05 were considered statistically significant. All figures were generated using ggplot2 v3.4.0 [39].

## Results

### Host morphological adaptations for durophagy

**Fig. 1:**
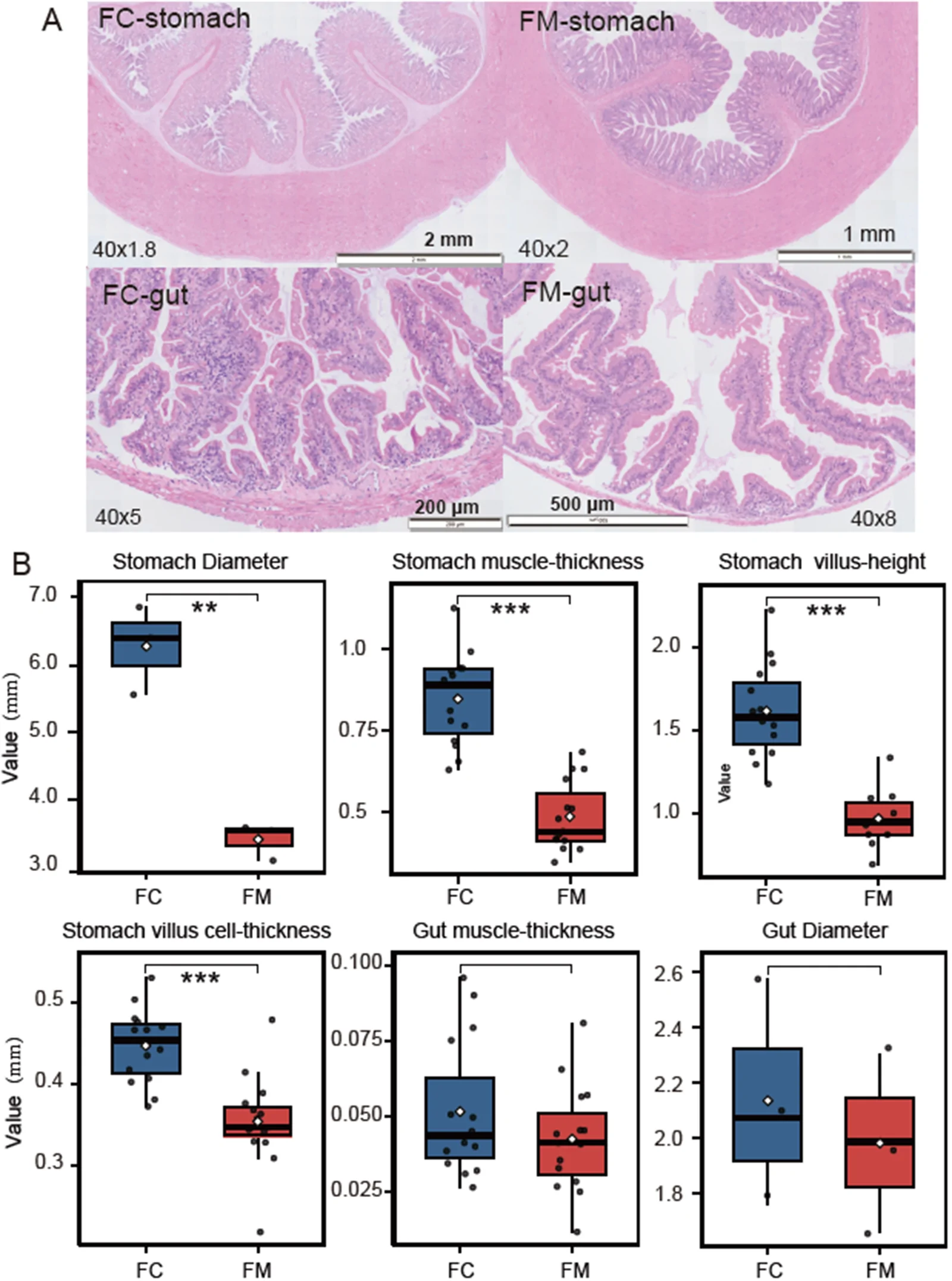
Morphological adaptations of the digestive tract in *F. cancrivora* (FC) and *F. multistriata* (FM). (**A**) Hematoxylin and eosin (H&E)-stained sections of gastric and gut tissues. (**B**) Quantification of gastric muscle thickness, gut villus height, and epithelial cell height (mean ± standard error of the mean; *n* = 3 per species; *p < 0.05, **p < 0.01, ***p < 0.001; Student’s t-test)

Histological analysis revealed pronounced tissue-specific morphological differences in the digestive system of *F. cancrivora* (FC) compared to its freshwater congener *F. multistriata* (FM) (Fig. 1A-B; Supplementary Table 1). In the stomach, FC exhibited significantly enlarged anatomical features associated with mechanical processing of hard prey. The stomach diameter was substantially larger in FC (6.268 ± 0.657 mm) than in FM (3.400 ± 0.266 mm). More notably, the gastric muscularis layer was significantly thickened in FC (0.849 ± 0.138 mm) compared to FM (0.488 ± 0.105 mm; Student’s t-test, *p* < 0.001), stomach villus height was markedly greater in FC (1.618 ± 0.285 mm) than in FM (0.966 ± 0.179 mm), accompanied by a thicker villous epithelial cell layer (FC: 0.447 ± 0.045 mm; FM: 0.354 ± 0.055 mm). Indicating enhanced capacity for mechanical breakdown of chitin-rich crab exoskeletons.

In the small intestine, no significant differences were detected between species in the thickness of the intestinal muscularis layer or in the cross-sectional diameter of the intestine (*p* > 0.05). Notably, when normalized to body length, FC possessed a significantly longer intestine relative to FM (Supplementary Fig. 1), suggesting an expanded absorptive surface that may facilitate nutrient extraction from crustacean prey.

### Host-driven chitinolytic capacity: gene expansion and enhanced activity

**Fig. 2:**
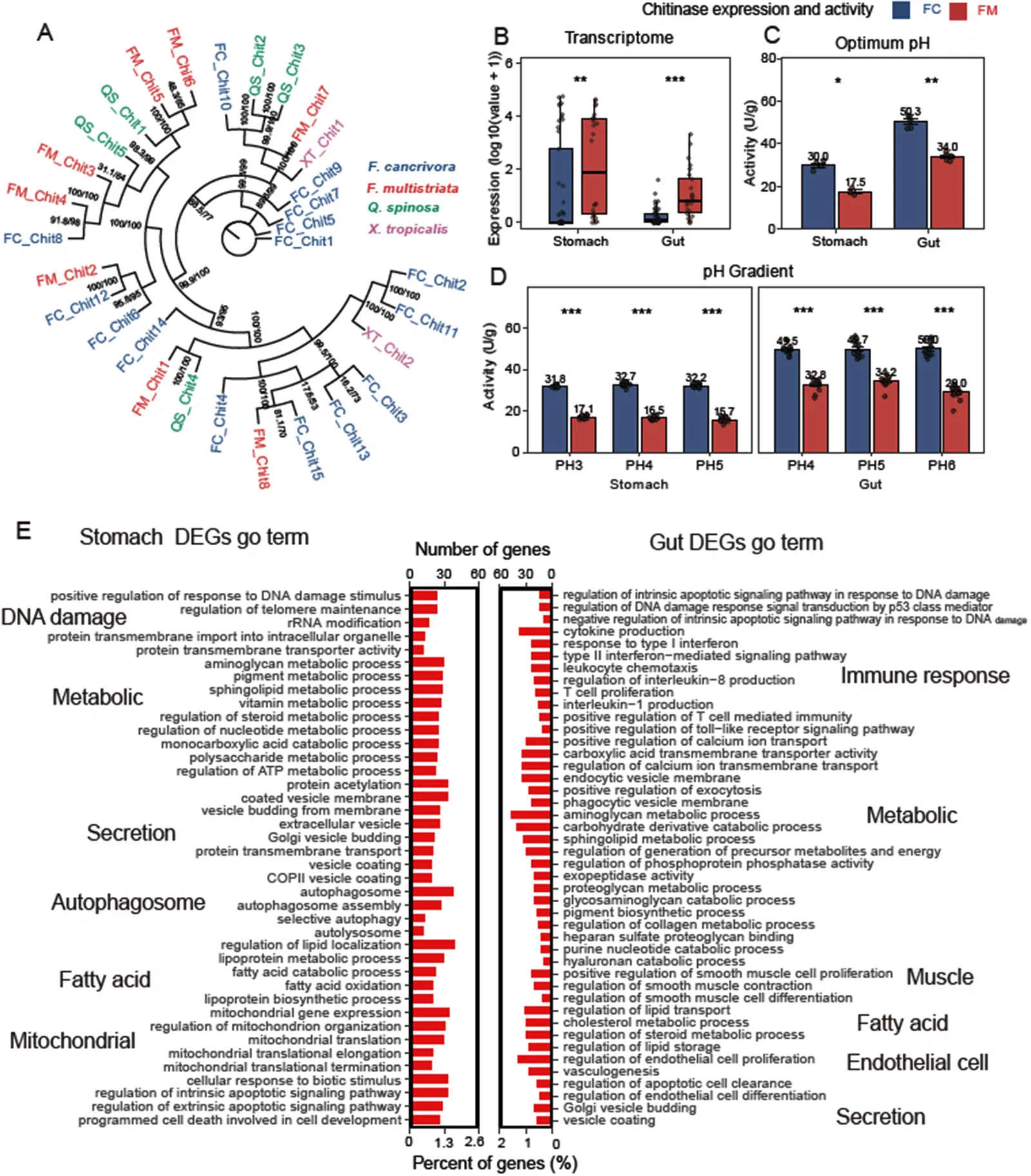
Putative chitinase-encoding transcripts and enzyme activity in different tissues, and gene ontology (GO) enrichment of differentially expressed genes (DEGs) between FC and FM. (**A**) Unrooted phylogenetic tree of chitinase transcripts from FC (blue), FM (red), *Q. spinosa* (QS, green), and *X. tropicalis* (XT, pink), branch support values at each node are shown as SH-aLRT (%) / Ultrafast Bootstrap (%). (**B**) Comparison of cumulative expression levels (transcript abundance of putative chitinase-encoding transcripts) between FC (blue) and FM (red) in gastric and gut tissues (*p* < 0.001; Student’s t-test). (**C**) Comparison of total chitinase enzymatic activity between FC (blue) and FM (red) in gastric and gut tissues. (**D**) Chitinase activity (U/g tissue) in the gastric tissue of FC (blue) and FM (red) under pH 3, pH 4, and pH 5 conditions. (**E**) Chitinase activity (U/g tissue) in the gut tissue of FC (blue) and FM (red) under pH 4, pH 5, and pH 6 conditions. (**F**) Gene Ontology (GO) functional enrichment analysis of differentially expressed genes (DEGs) upregulated in FC relative to FM. The x-axis represents the proportion of genes relative to the total orthologous gene set

Phylogenomic analysis of putative chitinase encoding transcripts across four amphibian species revealed a significant expansion of the putative chitinase-encoding transcript repertoire in FC. We identified 15 distinct putative chitinase-encoding transcripts in FC, compared to only 8 in FM, 5 in *Q. spinosa* (QS), and 2 in *X. tropicalis* (XT) (Fig. 2A). Instead of forming a single, monophyletic FC-specific clade, the 15 FC chitinase transcripts were distributed across multiple divergent clades, frequently clustering with orthologs from FM and other dicroglossid species (Fig. 2A). This scattered phylogenetic distribution suggests that the expanded chitinase repertoire in FC arose through multiple independent duplication events affecting distinct ancestral genes, rather than repeated tandem duplication of a single ancestral copy.

Transcriptomic analysis revealed an unexpected discordance between putative chitinase-encoding transcript repertoire size and transcripts abundance levels. Despite possessing nearly twice as many chitinase encoding transcripts as FM, FC exhibited lower average transcript abundance of individual chitinase encoding transcripts in both stomach and intestinal tissues. However, analysis of total chitinase transcripts abundance revealed a tissue-specific pattern (Fig. 2B). In the stomach, the cumulative abundance level was significantly higher in FC (FPKM = 77,896) than in FM (FPKM = 61,156; *p* < 0.05, Supplementary Fig. 2). Conversely, in the intestine, total chitinase transcripts abundance in FC was markedly lower (FPKM = 28) than in FM (FPKM = 1,227), although this difference did not reach statistical significance (*p* > 0.05).

Despite this mixed transcriptional profile, chitinase enzyme activity assays revealed a consistently superior functional capacity in FC. Under neutral pH conditions, FC exhibited significantly higher chitinase activity than FM in both the stomach (30.00 ± 1.89 vs. 17.56 ± 1.61 U/g; *p* < 0.001) and the intestine (50.37 ± 2.91 vs. 34.00 ± 1.82 U/g; *p* < 0.001) (Fig. 2C-D). Furthermore, across physiologically relevant pH gradients (stomach: pH 3.0–5.0; intestine: pH 4.0–6.0), FC maintained significantly higher chitinase activity in both digestive compartments (Fig. 2C-D). This broad pH tolerance suggests that FC chitinases are adapted to function effectively across the dynamic conditions of the digestive tract, from the acidic stomach to the near-neutral intestine.

Notably, a striking tissue-specific pattern emerged: in FC, intestinal chitinase activity exceeded gastric activity by approximately 68% across all tested pH values. This pattern contrasts sharply with that observed in other vertebrates (e.g., mice and crab-eating monkeys) [40, 41], where gastric chitinase activity predominates. The elevated and sustained chitinolytic capacity in the FC intestine may reflect an extended digestive processing strategy tailored to the slow breakdown of chitin-rich crab exoskeletons.

### Host transcriptional reprogramming reflects metabolic and stress responses

Differential expression analysis between FC and FM identified 2387 and 3099 DEGs in the stomach and gut, respectively. In the FC stomach, upregulated genes were enriched for mitochondrial respiration (GO:0140053), ATP metabolism (GO:1903578), and polysaccharide metabolic processes (GO:0005976), reflecting the high energy demand of processing chitin-rich prey (Fig. 2E). Concurrently, enrichment of DNA damage response (GO:2001022) and autophagy (GO:0061912) pathways suggested a response to oxidative stress from high-protein metabolism and mechanical abrasion (Supplementary Table 2).

In the FC gut, upregulated genes were enriched for lipid transport (GO:0032368) and cholesterol metabolism (GO:0019218), aligning with the high-lipid content of crustaceans, as well as pathways for DNA repair (GO:1902229) and immune modulation (e.g., interleukin production and T-cell proliferation), indicating a robust defense against both oxidative and pathogenic stressors (Supplementary Table 2).

### Gut microbiota: distinct community structure and functional specialization

**Fig. 3:**
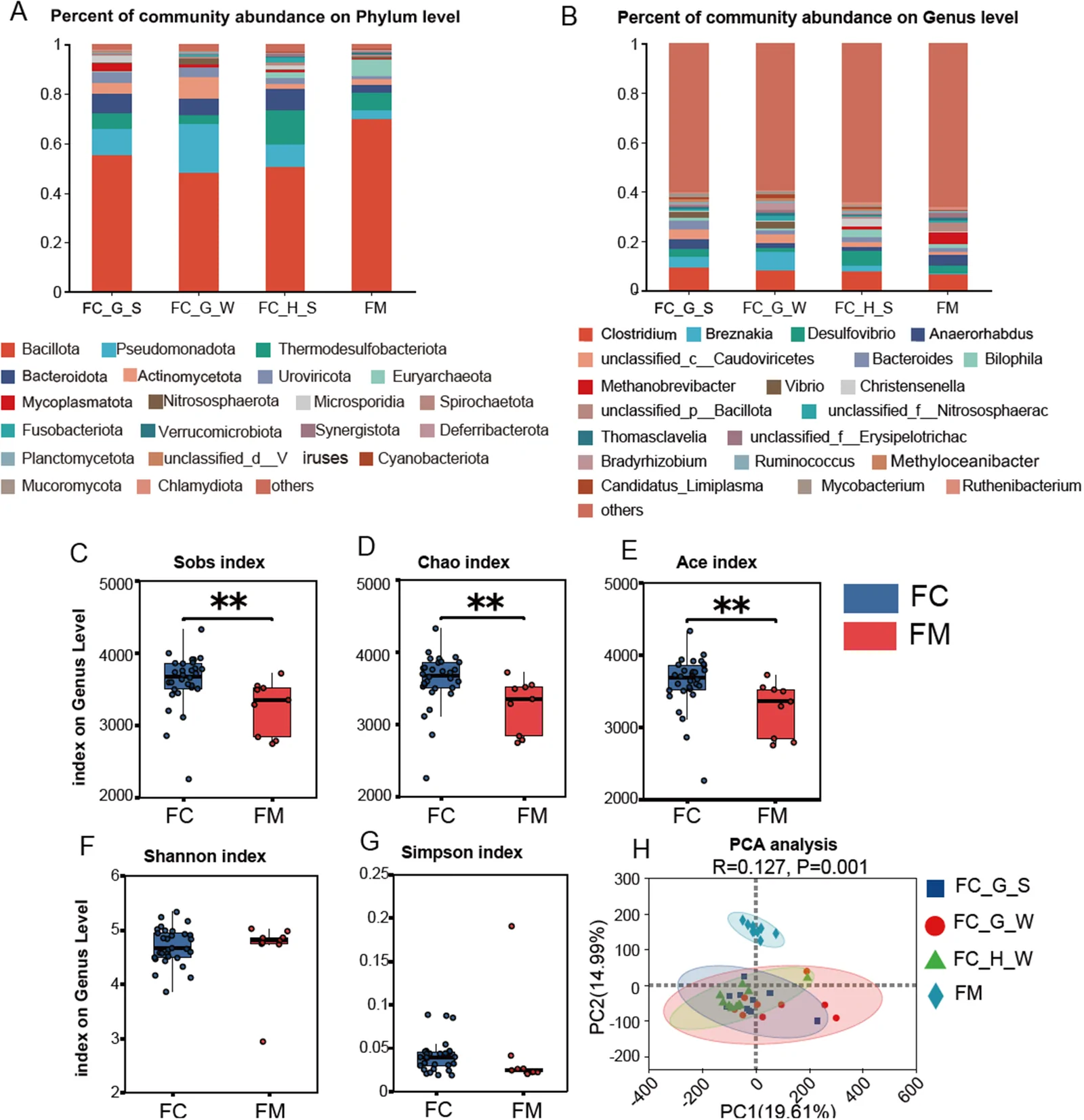
Comparison of gut microbial community characteristics between fejervarya cancrivora (FC) and fejervarya multistriata (FM). (**A**) The relative abundance of the top microbial phylum in the gut across different groups. (**B**) Differential microbial genera between FC and FM. (**C-G**) Alpha-diversity analysis of the gut microbiota. Box plots depict the differences in: (**C**) Sobs index, (**D**) Chao index, (**E**) Ace index (species richness), (**F**) Shannon index, and (**G**) Simpson index (species diversity) between FC and FM. (*P < 0.05, **P < 0.01, ***P < 0.001). (**H**) Principal Component Analysis (PCA) plot illustrating β-diversity

Metagenomic sequencing revealed marked differences in the gut microbial communities of FC and FM (Supplementary Table 3). FC harbored a gut microbiota with significantly higher species richness (Chao1, ACE indices; *p* < 0.05) compared to FM, but with similar evenness (Shannon index; Fig. 3C-G). Beta diversity analysis based on Bray-Curtis dissimilarity revealed a clear and significant separation between FC and FM communities (ANOSIM, R² = 0.127, *p* < 0.01, Fig. 3H). Hierarchical clustering analysis further confirmed this divergence, with all FC samples forming a distinct clade separate from FM (Supplementary Fig. 3). Notably, FC samples collected from the Gaoqiao population across different seasons clustered tightly together (Fig. 3H), indicating minimal seasonal variation in gut microbial community composition. This temporal stability suggests a host-selected microbial community adapted to maintain consistent digestive function despite fluctuations in prey availability within the intertidal environment.

At the phylum level, Bacillota (Firmicutes) dominated both species but was relatively less abundant in FC (51.3%) than in FM (70.0%) (Fig. 3A, Supplementary Table 4). Other prevalent phyla among the top five in both species included Thermodesulfobacteriota, Pseudomonadota (Proteobacteria), and Bacteroidota. This phylum-level composition is broadly consistent with previous reports in other amphibian species [6, 7, 42].

Despite these similarities at the phylum level, marked differences emerged at finer taxonomic resolution. At the genus level, both species shared dominant taxa including *Clostridium* and *Breznakia*. However, differential abundance analysis (Wilcoxon rank-sum test) revealed distinct signature genera linked to functional specialization (Fig. 3B; Supplementary Tables 4–5). FC exhibited significantly higher relative abundances of taxa associated with complex nutrient metabolism and short-chain fatty acid production, including *Breznakia* (*p* < 0.01, FDR < 0.05), *Alistipes* (*p* < 0.001, FDR < 0.05), *Paraclostridium* (*p* < 0.01, FDR < 0.05), *Rikenella* (*p* < 0.01, FDR < 0.05) and *Massilimicrobiota* [43] (*p* < 0.05, FDR < 0.05), and marine species *Vibrio* (*p* < 0.01, FDR < 0.05) [44]. In contrast, FM was enriched in genera linked to polysaccharide fermentation (*Blautia*) [45], and taxa often associated with variable or opportunistic environments (*Candidatus Limipiscum*, *Faecalicatena*).

Collectively, these results indicate that while FC and FM share core microbial lineages, FC harbors a gut microbiota with greater taxonomic richness and a distinct community structure enriched for taxa involved in energy harvest from lipid-rich diets. The reduced seasonal variability observed in the FC population further suggests a stable, host-selected microbial community adapted to the fluctuating intertidal environment.

### Functional divergence in microbial metabolic potential

Despite the chitin-rich diet of FC, Carbohydrate-Active Enzyme (CAZyme) profiling revealed its microbiota exhibited significantly lower abundance of chitinase-encoding genes from the GH18 family compared to FM (FC: 7.3 ± 0.9 RPKM; FM: 12.6 ± 1.5 RPKM; *p* < 0.01; Supplementary Fig. 4). This finding aligns with the hypothesis that FC relies primarily on host-derived chitinases for chitin digestion, whereas FM depends more heavily on microbial chitinolytic activity. Conversely, FC’s microbiota was significantly enriched in KEGG pathways related to stress tolerance and energy efficiency, including DNA repair (ko03430), fatty acid metabolism (ko01212), and oxidative phosphorylation (ko00190) (Supplementary Table 6).

**Fig. 4:**
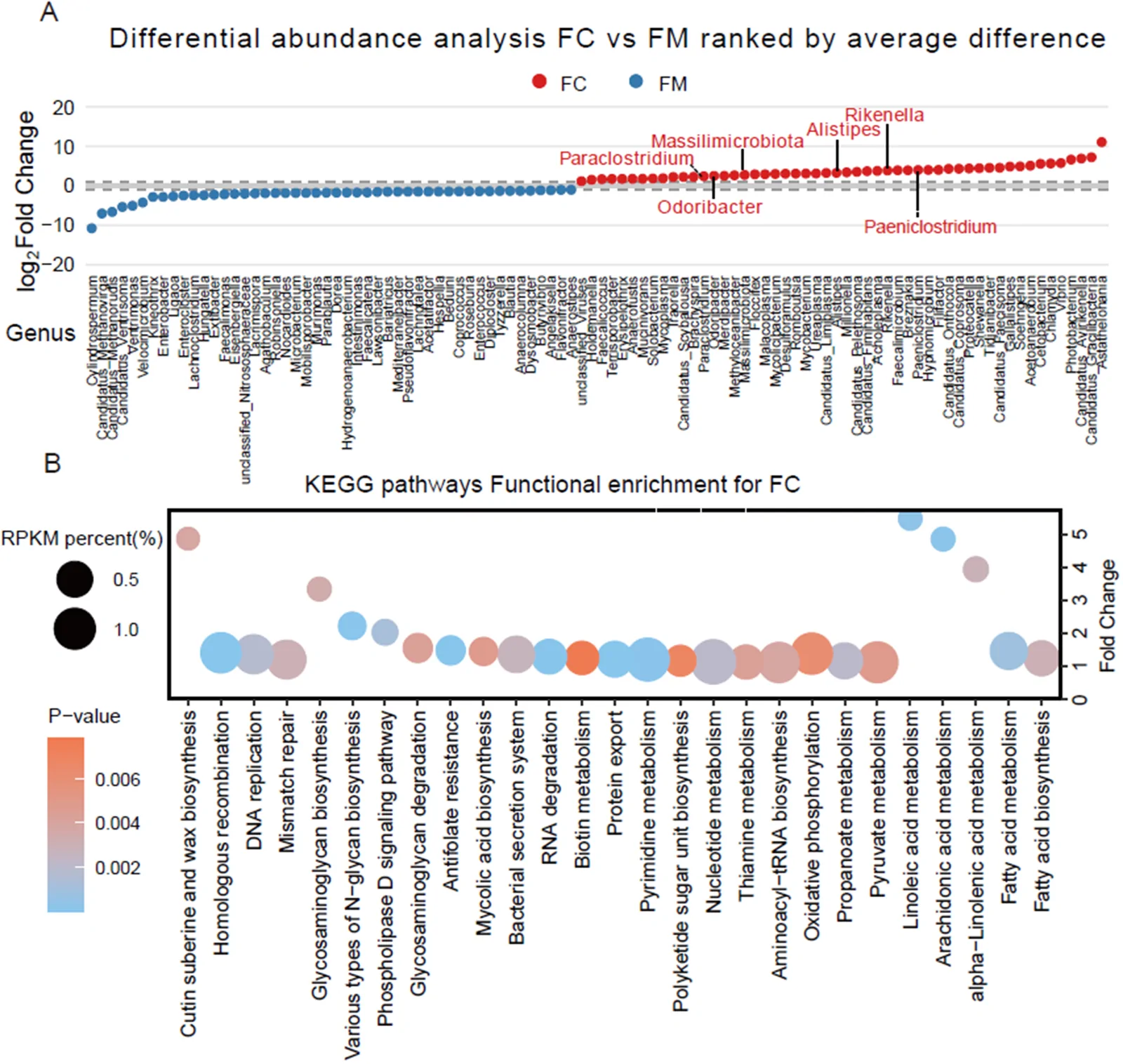
Taxonomic and functional alterations in the gut microbiome for FC and FM. **A**, Differential abundance analysis of the top 100 genera between the FC and FM groups.The plot displays the fold change of genera ranked by average difference. Red and blue dots denote genera enriched in the FC and FM groups, respectively. Dashed lines represent the significance threshold. Key discriminative genera, including *Rikenella*, *Alistipes*, and *Paeniclostridium*, are closely related to metabolism in literature. **B**, Functional enrichment analysis of the metagenome based on KEGG pathways. Bubble size is proportional to the relative abundance (RPKM percentage), and the color gradient represents the P-value. The y-axis indicates the fold change of the respective functional pathways

Further pathway enrichment analysis confirmed that FC’s microbiota was significantly enriched in three key categories (Fig. 4B, Supplementary Table 6): (1) lipid metabolism pathways (e.g., fatty acid biosynthesis, linoleic acid metabolism, arachidonic acid metabolism, α-linolenic acid metabolism), 2) energy metabolism pathways (e.g., oxidative phosphorylation, pyruvate metabolism, propionate metabolism), and 3) nucleotide maintenance pathways (e.g., mismatch repair, DNA replication, homologous recombination). The marked enrichment of DNA repair pathways suggests a microbial adaptation to the high-salinity, ion-rich marine environment, where osmotic stress and reactive oxygen species can induce DNA damage [46].

### Diet and environment differentially shape microbial functional profiles

To identify the microbial taxa driving these functional differences, we integrated differential abundance analysis (|log2FC| > 1, *p* < 0.05) with known functional roles from the literature (Fig. 4A; Supplementary Table 5). FC was enriched in taxa associated with metabolic optimization and stress adaptation, including *Alistipes* (uric acid metabolism) [47], *Breznakia* (lipid utilization in specialized predators) [16, 48], *Rikenella* and *Paeniclostridium* (inflammatory regulation and adaptation to arid environments) [49, 50], *Methyloceanibacter* (adaptation to marine environments) [51]. These taxa likely enhance FC’s ability to extract energy from high-protein, lipid-rich crustacean prey and tolerate the multifaceted stressors of the intertidal habitat.

To further disentangle the relative contributions of diet and salinity to the observed microbial functional profiles, we conducted a controlled feeding experiment. Wild-caught FC were maintained in seawater from their native habitat and fed a cricket diet for four weeks (replacing the natural crab diet while preserving the saline environment, Supplementary Fig. 5, Supplementary Table 7). Functional enrichment analysis revealed that pathways related to lipid metabolism (e.g., fatty acid biosynthesis) and oxidative phosphorylation were significantly enriched in wild, crab-fed FC compared to laboratory, cricket-fed individuals. In contrast, no significant differences were observed between the two groups in pathways associated with DNA damage repair (mismatch repair). These findings demonstrate that the enrichment of lipid metabolism and energy production pathways is a plastic response driven by the high-lipid crab diet, whereas the enhanced DNA repair capacity represents a stable adaptation to the high-salinity environment that persists regardless of dietary composition.

### Integrative host-microbiome co-adaptation

**Fig. 5:**
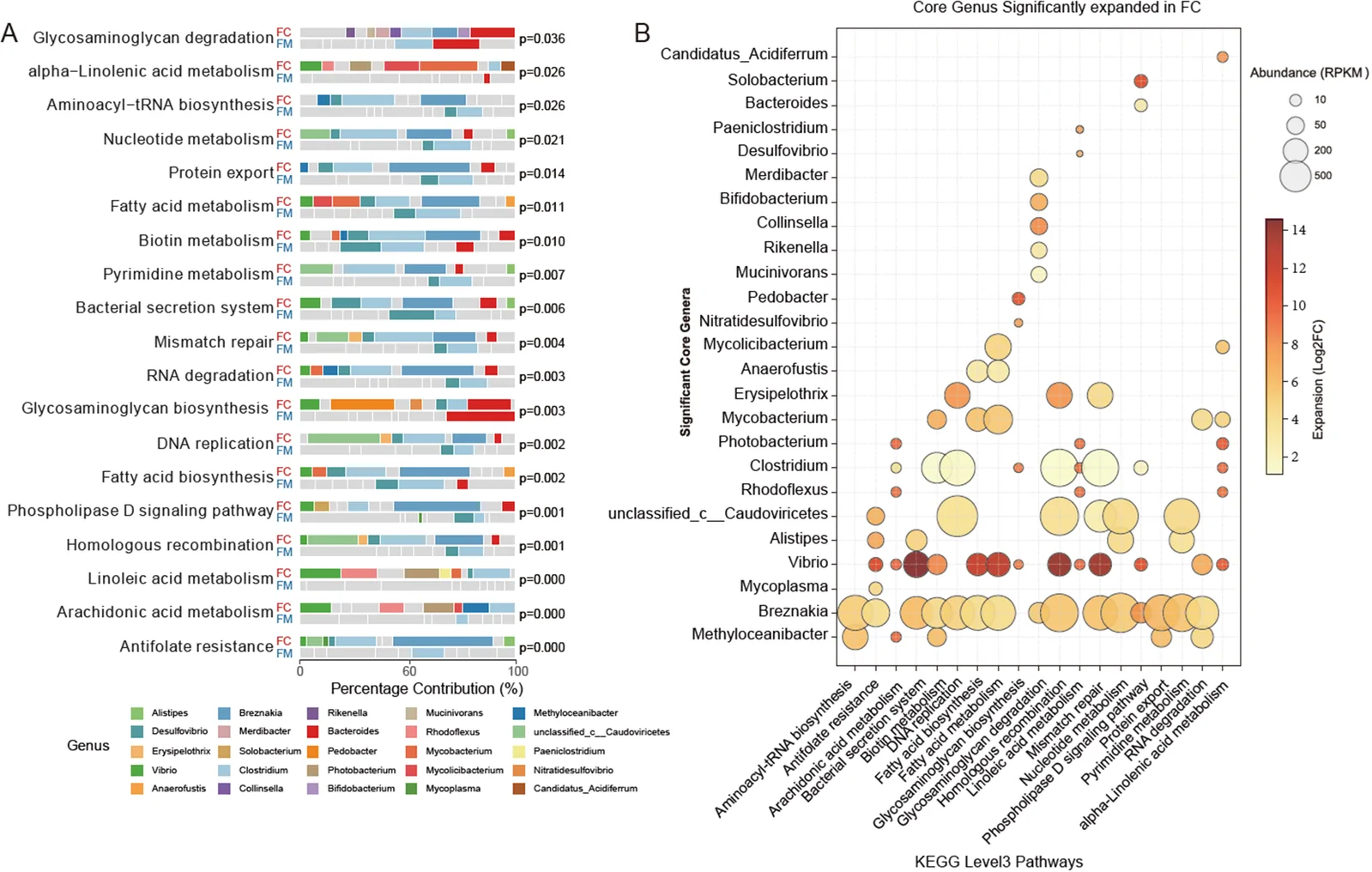
Taxonomic contribution to functional alterations and expansion of core genera in the FC group. **A**, Stacked bar charts showing the percentage contribution of distinct bacterial genera to the abundance of differentially enriched KEGG metabolic pathways. For each pathway, paired bars represent the FC (top) and FM (bottom) groups. Colors indicate core genera as defined in the legend (top 10 abundance rpkm for each KEGG pathway). Wilcoxon test P-values listed on the right denote the statistical significance of the difference between FC and FM. **B**, Bubble plot illustrating the specific core genera significantly expanded in the FC group across associated KEGG Level 3 pathways. The y-axis represents significant core genera, and the x-axis displays the corresponding metabolic pathways. The size of each circle is proportional to the mean abundance (RPKM), while the color gradient indicates the magnitude of expansion (Log2fold change) in the FC group relative to the FM group

The functional partitioning was further supported by taxonomic contribution analysis (top 10 contributors per pathway). We identified 25 “core genera” that were both functionally dominant and significantly expanded in FC (Log2FC > 1, Wilcoxon rank-sum test, *P* < 0.05; Fig. 5A-B and Supplementary Tables 8–9). Notably, *Breznakia* emerged as a key multifunctional taxon, contributing substantially to multiple enriched metabolic pathways. Finally, Pearson correlation analysis revealed significant positive correlations between host and microbial pathways, suggesting a coordinated molecular dialogue. For instance, host pathways for lipid transport were strongly correlated with microbial fatty acid biosynthesis (*r* > 0.95, *p* < 0.001), and host DNA damage response genes were correlated with microbial mismatch repair mechanisms (*r* > 0.85, *p* < 0.001) (Table 1). This synchronization reinforces the model of a holobiont-level response to the dual pressures of a high-lipid diet and a genotoxic, high-salinity environment.

**Table 1:** host gut DEGs and gut microbiome correlation

FC gut DEGs	Meta Differential abundance	Correlation coefficient	*P*-value
Regulation of collagen metabolic process	Glycosaminoglycan biosynthesis - chondroitin sulfate / dermatan sulfate	0.9367	< 0.001
Heparan sulfate proteoglycan binding	Glycosaminoglycan biosynthesis - heparan sulfate / heparin	0.8833	< 0.001
Regulation of lipid transport	Arachidonic acid metabolism	0.9875	< 0.001
Sphingolipid metabolic process	Fatty acid biosynthesis	0.9793	< 0.001
Regulation of generation of precursor metabolites and energy	RNA degradation	0.956	< 0.001
Cytokine production	Bacterial secretion system	0.694	< 0.001
Regulation of interleukin-8 production	Bacterial secretion system	0.7455	< 0.001
Regulation of generation of precursor metabolites and energy	Oxidative phosphorylation	0.7987	< 0.001
Regulation of DNA damage response signal transduction by p53 class mediator	DNA replication	0.8767	< 0.001
Negative regulation of intrinsic apoptotic signaling pathway in response to DNA damage	Mismatch repair	0.8587	< 0.001

## Discussion

Our integrated analysis reveals that the successful invasion of the marine intertidal niche by *Fejervarya cancrivora* (FC) is underpinned by a synergistic host-microbe functional partitioning. We demonstrate that the host has evolved morphological and enzymatic adaptations to serve as the primary driver of chitin digestion, while the gut microbiota specializes in downstream nutrient utilization, particularly lipid metabolism. Furthermore, both the host and its microbiome exhibit convergent adaptations to high-salinity stress through enhanced DNA damage repair pathways. A controlled feeding experiment confirmed that the microbial lipid metabolism is a diet-driven response, while the enhanced DNA repair pathways represent a stable adaptation to the saline environment. This synergistic strategy, operating at the holobiont level, provides a new framework for understanding how stress-vulnerable organisms like amphibians can overcome the dual barriers of extreme diets and environments.

### The host as the primary agent of chitin digestion

The digestive system of FC exhibits a suite of morphological and molecular adaptations that position the host as the primary driver of chitin digestion, a strategy fundamentally distinct from the microbe-dependent chitin degradation observed in its freshwater congener. At the morphological level, FC displays classic adaptations to a durophagous diet, including a thickened gastric muscularis and elongated intestinal villi, features analogous to those observed in other hard-prey specialists such as red knots [52, 53]. These modifications reflect the high mechanical demands imposed by a diet in which crustaceans constitute over 60% of intake [4], presenting a significantly higher chitin burden than the soft-bodied invertebrates typical of freshwater amphibians [7, 54–56].

Complementing these physical adaptations, FC possesses a markedly expanded endogenous chitinolytic system. The chitinase gene family in FC (15 non-redundant putative transcripts) is nearly twice the size of that in FM (8 non-redundant putative transcripts), aligning with the evolutionary trajectory observed in insectivorous mammals that retain multiple functional *CHIA* copies to digest arthropod exoskeletons [40, 57, 58]. This expansion likely resulted from gene duplication events driven by natural selection for dietary specialization [59, 60]. Functional assays confirm that this expanded gene repertoire translates to enhanced digestive capacity. FC exhibits significantly higher chitinase activity than FM across both stomach and intestinal tissues and maintains this superiority across a broad pH range (pH 3.0–6.0), reflecting adaptation to the dynamic chemical environment of the digestive tract.

A seemingly paradoxical finding emerged: despite lower average transcript abundance of individual chitinase-like transcripts, FC displayed significantly higher functional enzyme activity. This discrepancy points to potent post-transcriptional or post-translational regulation. We hypothesize three non-mutually exclusive explanations: (1) enhanced translational efficiency or protein stability of FC chitinases under acidic and high-ion conditions; (2) the presence of novel, high-activity isoforms among the expanded gene family that were not fully captured by transcript abundance metrics; or (3) ion-mediated activation of chitinase enzymes by the high salinity of the gut lumen, a mechanism known for some halotolerant enzymes [54]. Most strikingly, FC exhibited a unique tissue-specific pattern wherein intestinal chitinase activity exceeded gastric activity by approximately 18%, a reversal of the typical vertebrate pattern observed in FM, mice, and crab-eating monkeys [40, 41]. This unusual feature may reflect an extended digestive processing strategy tailored to the slow, gradual breakdown of large, tough crab fragments, necessitating sustained chitinolytic capacity throughout the intestine. Together, these morphological, genetic, and enzymatic adaptations establish FC as the primary agent of chitin digestion, efficiently unlocking the protein and energy-rich contents of its crustacean prey.

While the pronounced gastric thickening and expanded chitinase transcripts in FC highlight its extreme dietary specialization, these traits likely represent a remarkable case of exaptation rather than de novo innovations. Morphological data from other dicroglossid frogs reveal a naturally robust ancestral baseline for gastric muscularis thickness (e.g., 390–742 μm in *Quasipaa* and *Nanorana*, Supplementary Table 1) capable of processing hard-shelled prey, within which the freshwater FM (~ 488 μm) naturally falls. Similarly, at the molecular level, the retention of 8 distinct chitinase transcripts in FM, coupled with the scattered phylogenetic distribution of FC sequences, indicates that the extreme expansion in FC (to 15 transcripts) arose through multiple independent duplications of an already diverse ancestral repertoire. Ultimately, while this pre-adaptation model parsimoniously reconciles the morphological and molecular data, definitively mapping the precise evolutionary trajectory of these durophagous traits will require future comparative studies incorporating broader phylogenetic sampling of the *Fejervarya* genus.

### The microbiota as a specialist in lipid metabolism and stress resilience

In stark contrast to the host’s dominance in chitin digestion, the gut microbiota of FC has evolved a complementary functional specialization. While its freshwater relative FM exhibits high microbial chitinase potential (GH18 abundance), FC’s microbiota shows significantly lower abundance of chitinase-encoding genes, indicating a diminished role in chitin breakdown. Instead, the FC microbiome is functionally specialized for tasks that directly complement host capabilities: lipid metabolism and stress resilience.

The enrichment of lipid metabolism pathways, including fatty acid biosynthesis, linoleic acid metabolism, and oxidative phosphorylation, represents a direct functional response to the high-lipid content of crustacean prey. This pattern mirrors adaptations observed in other dietary specialists, such as the snail-eating snake *Pareas menglaensis*, which harbors a microbiome enriched in lipid-extraction capabilities to overcome the energetic constraints of its lipid-poor diet [16], and the giant panda, whose microbiota compensates for a carnivorous digestive system by enriching starch metabolism pathways to process bamboo [17]. Our transplant experiment provides causal evidence for this link: when wild-caught FC were switched from a crab to a cricket diet, the enrichment of lipid metabolism and oxidative phosphorylation pathways was abolished, confirming their dependence on dietary lipid intake. Key genera enriched in FC, including *Breznakia* and *Alistipes*, are likely central to this process. *Breznakia* has been demonstrated to serve as a critical metabolic symbiont in multiple animal species, contributing substantially to energy harvest through short-chain fatty acid production [48, 61, 62]. *Alistipes* is known to enhance uric acid metabolism [47], potentially aiding in nitrogen waste processing and indirectly optimizing water balance in saline environments.

Even more striking than the metabolic specialization is the stable enrichment of microbial DNA repair pathways, including mismatch repair, homologous recombination, and DNA replication. The high salinity of the intertidal zone is a potent source of osmotic and oxidative stress, known to induce DNA damage across all cell types [63]. We propose that the enhanced capacity for DNA repair within the gut microbial community represents a critical adaptation for holobiont survival, enabling a robust and functional microbial consortium to persist under constant genotoxic stress. The controlled feeding experiment was instrumental in establishing the nature of this adaptation: unlike the diet-sensitive lipid metabolism pathways, DNA repair enrichment persisted unchanged in laboratory-fed FC maintained in seawater, confirming it is a hard-wired adaptation to the saline environment rather than a dietary artifact. This finding resonates with studies in other extreme environments, such as deserts, where the gut microbiome buffers osmotic stress for host organisms like *Meriones unguiculatus* [49]. The strong positive correlation between host DNA damage response genes and microbial DNA repair pathways (Table 1) further suggests a coordinated, two-pronged strategy to maintain genomic integrity across the entire holobiont in response to a shared environmental stressor, a mechanism previously undocumented in amphibians.

Furthermore, our study suggests a potentially underappreciated role of the microbiome in FC osmoregulation. Given that *Alistipes* enhances uric acid metabolism in mouse models [47], it may assist FC in processing nitrogenous waste more efficiently, thereby indirectly optimizing water balance in saline environments. Analogous to the mechanism in desert rodents where probiotic-produced short-chain fatty acids modulate Na⁺/H⁺ exchange and promote water absorption [49], the enrichment of SCFA-producing genera like *Breznakia* in FC may perform a similar physiological function. This comprehensive adaptation capacity, addressing both digestive and osmotic challenges, allows FC to thrive in environments where other amphibians struggle to survive.

### The holobiont model for marine adaptation

These findings delineate a dual strategy model within the FC holobiont. Regarding dietary challenges, there is a clear functional division of labor: the host assumes responsibility for the primary digestive challenge, that of mechanical and chemical breakdown of chitin-rich prey, through morphological reinforcement and an expanded, highly active endogenous chitinase system while the microbiota provides complementary metabolic capabilities by maximizing energy extraction from the lipid-rich components of the diet, a function that exhibits plasticity in response to dietary composition.

However, in response to the sustained osmotic and oxidative stress of the intertidal zone, the holobiont exhibits striking functional convergent adaptation to the high-salinity environment. Rather than a division of labor, both the host and its diverse microbial constituents have independently enriched their respective DNA repair pathways to protect their own genomes. This division and convergence strategy enables FC to effectively utilize challenging food resources while thriving under genotoxic stress, occupying an extreme niche where few amphibians can survive.

**Fig. 6:**
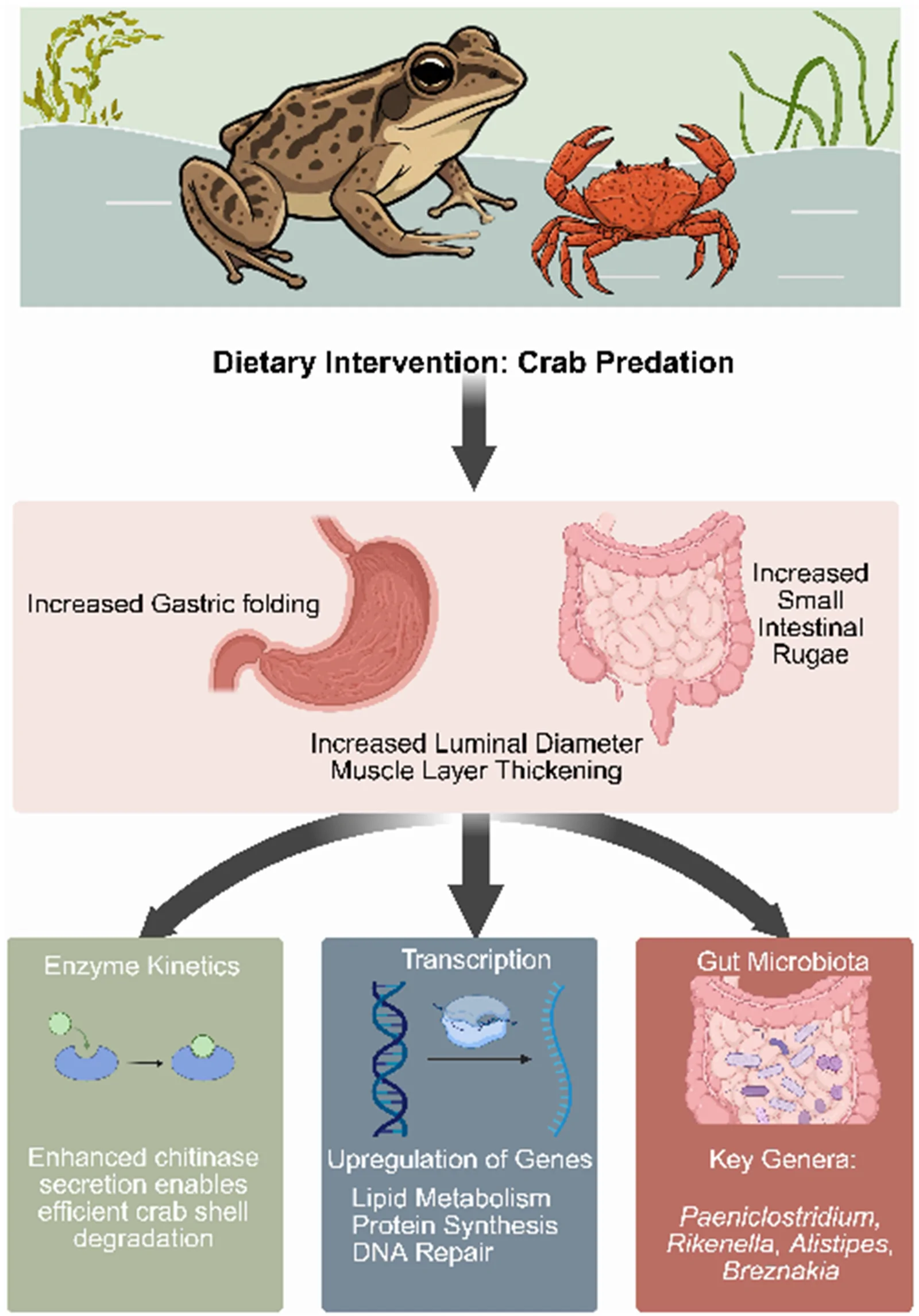
Holobiont-level adaptations driving crab diet specialization in *F. cancrivora*. The schematic illustrates coordinated responses across morphological remodeling, enhanced chitinase kinetics, transcriptional reprogramming, and microbiome functional shifts enabling marine niche colonization

Our findings significantly expand the understanding of amphibian marine adaptation. Previous models focused solely on the host’s osmoregulatory physiology [12, 13]. We propose a more comprehensive “holobiont adaptation model” (Fig. 6), where the host’s genetic and morphological toolkit solves the primary digestive challenge, while the microbiome provides flexible, fast-evolving metabolic capabilities. Crucially, this model also incorporates the parallel adaptive responses of both entities to environmental stress. The stability of the FC microbial community across seasons further underscores its co-evolved, non-random assembly. This case study adds to a growing body of literature suggesting that successful colonization of extreme niches, from high altitudes [64, 65] to subterranean habitats [66], often hinges on synergistic host-microbe interactions. The cross-species functional convergence in DNA repair pathways provides compelling evidence that extreme environmental selective pressures act simultaneously on both host and symbiont genomes, forcing the co-evolution of molecular mechanisms to maintain genomic stability, a principle that may apply broadly to vertebrate expansion into extreme habitats.

### Limitations and future directions

This study has several limitations. First, our estimation of chitinase transcriptome-inferred repertoire size (15 in FC vs. 8 in FM) relies on *de novo* transcriptome assembly. While phylogenetic clustering was used to mitigate redundancy, the lack of a high-quality reference genome prevents definitive exclusion of alternative splicing isoforms or assembly artifacts. Second, although the transplant experiment provides causal insight at the community function level, our findings remain partially correlative. Future work using gnotobiotic models is needed to establish causality for specific microbial taxa. For instance, colonizing germ-free FM with key FC microbes (e.g., *Breznakia*, *Alistipes*) and then challenging them with a high-salinity, high-crab diet would test the functional necessity of these taxa. Third, while species-specific divergence is evident, we cannot fully disentangle the influence of environmental microbiota from host-selected microbiome assembly without common-garden experiments comparing populations under identical conditions.

Building on these findings, we propose three actionable research directions: (1) Genomic validation: sequencing the chromosome-level genome of *F. cancrivora* is essential to map the *CHIA* gene locus and determine whether the expanded chitinase repertoire arose from tandem duplications or whole-genome duplication events. (2) Functional verification: controlled feeding experiments switching FM to a crab diet and FC to an insect diet would reveal the plasticity of these traits, helping determine whether the observed adaptations are obligate evolutionary traits or inducible responses to immediate dietary intake. (3) Metabolomic integration: integrating metabolomics with our current metagenomic data would provide direct evidence of functional metabolites produced by the microbiota. Identifying microbial osmolytes or short-chain fatty acids that aid in host osmoregulation would mechanistically link the microbiome to salinity tolerance. (4) Our reliance on a pairwise comparison restricts our ability to definitively trace the evolutionary origins of these traits; future comparative studies incorporating a broader phylogenetic sampling of congeneric *Fejervarya* genus species are required to resolve whether these extreme morphological and genetic features are *de novo* lineage-specific adaptations to the marine environment or amplifications of ancestral pre-adaptations.

## Conclusion

In conclusion, this study demonstrates that the marine adaptation of *F. cancrivora* is not merely a story of physiological tolerance, but a compelling example of host-microbe co-adaptation. Unlike many dietary specialists that rely primarily on microbial fermentation for digesting recalcitrant food (e.g., herbivores), we show that *F. cancrivora* retains the primary role in chitin degradation through host-driven adaptations: gastric hypertrophy and the expansion of acidic-active putative chitinase-encoding transcripts. The gut microbiota, conversely, plays a complementary rather than redundant role, specializing in lipid metabolism to maximize energy extraction from crustacean prey and DNA repair to mitigate the oxidative and osmotic stress of the intertidal habitat. By partitioning critical functions, chitin digestion to the host, and lipid metabolism and stress resilience to the microbiome, this amphibian has forged a successful strategy for conquering a uniquely challenging ecological niche. This framework refines our understanding of amphibian marine adaptation, suggesting that successful colonization of extreme environments requires not only physiological osmoregulation but also a coordinated digestive strategy where host and microbiome function as an integrated holobiont. More broadly, this synergistic adaptation model provides a powerful lens for future investigations into the evolution of vertebrate adaptations to extreme environments.

## Supplementary Information

Below is the link to the electronic supplementary material.

Supplementary Material 1

Supplementary Material 2

Supplementary Material 3

Supplementary Material 4

Supplementary Material 5

Supplementary Material 6

Supplementary Material 7

Supplementary Material 8

Supplementary Material 9

Supplementary Material 10

Supplementary Material 11

## Data Availability

Sequencing data have been uploaded to Genome Sequence Archive (https://ngdc.cncb.ac.cn/gsa/) with the accession numbers PRJCA049165.
