# Supplementary material for: Hologenomic rewiring facilitates dietary adaptation to chitin-rich marine resources in the crab-eating frog
Source: Front Zool. 2026 Jul 21;23:38. doi: 10.1186/s12983-026-00626-1 (PMC13673647; doi:10.1186/s12983-026-00626-1)
Supplement: Supplementary file 1 [file 12983_2026_626_MOESM1_ESM.pdf]

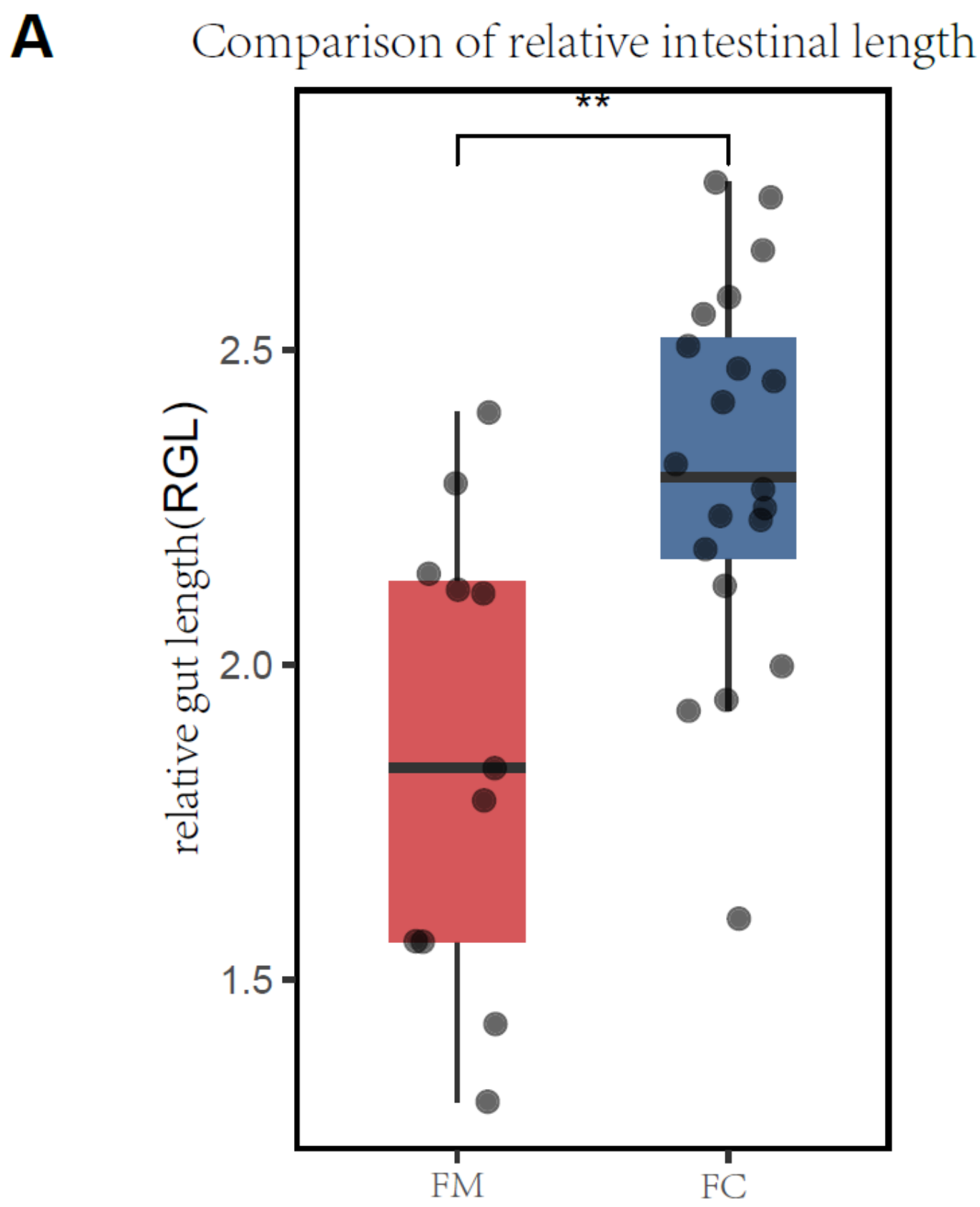

Supplementary Figure 1. Comparison of relative gut length (RGL) (gut length / body length): Box plot showing the RGL of FM and FC. The RGL of FC is significantly longer than that of FM (** P-value <0.01, T-test).

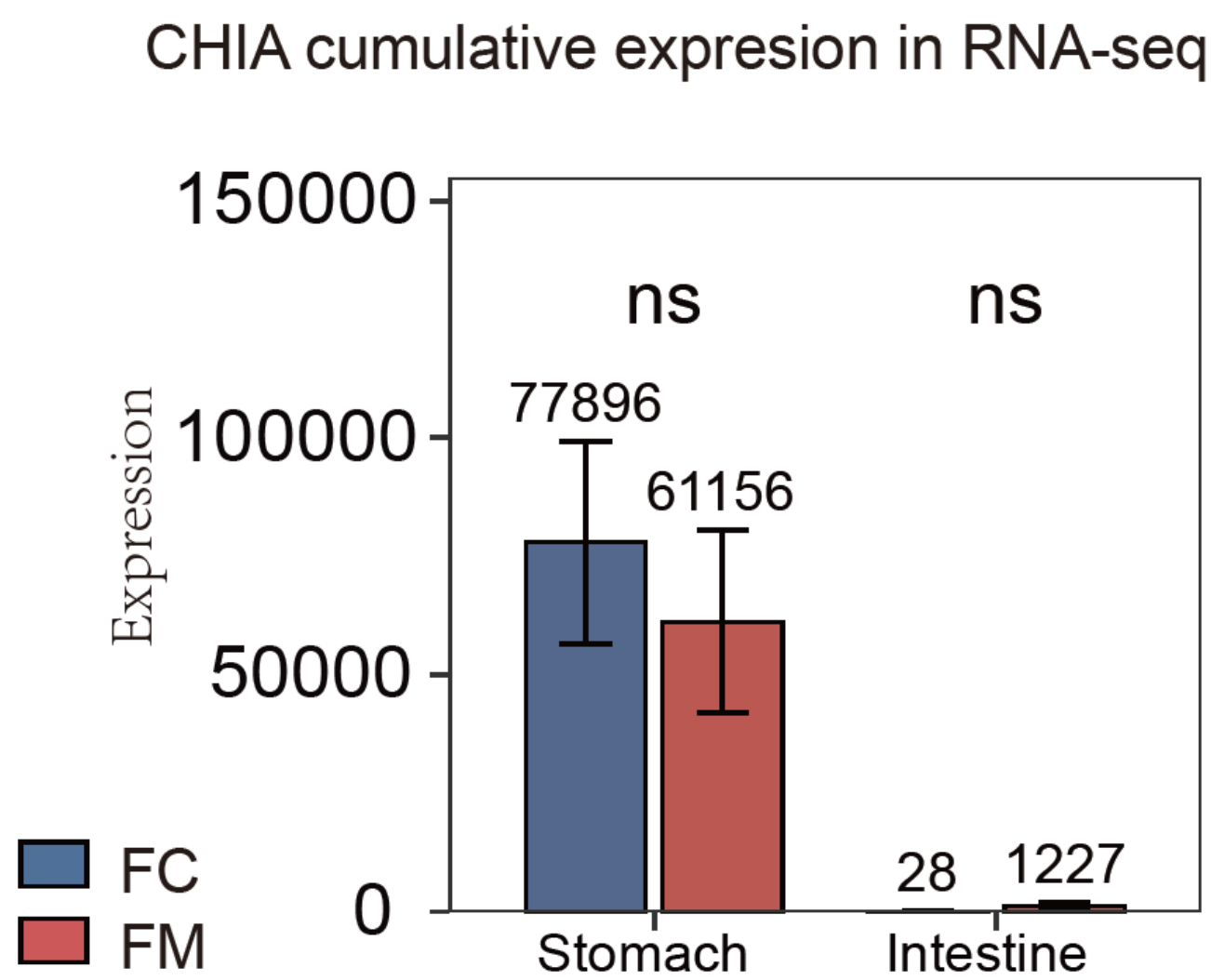

Supplementary Figure2. CHIA cumulative expression: RNA-seq data showing the cumulative expression of the CHIA gene in the stomach and intestine. There is no significant difference (ns,

Mann-Whitney U test) between FC and FM in either tissue.

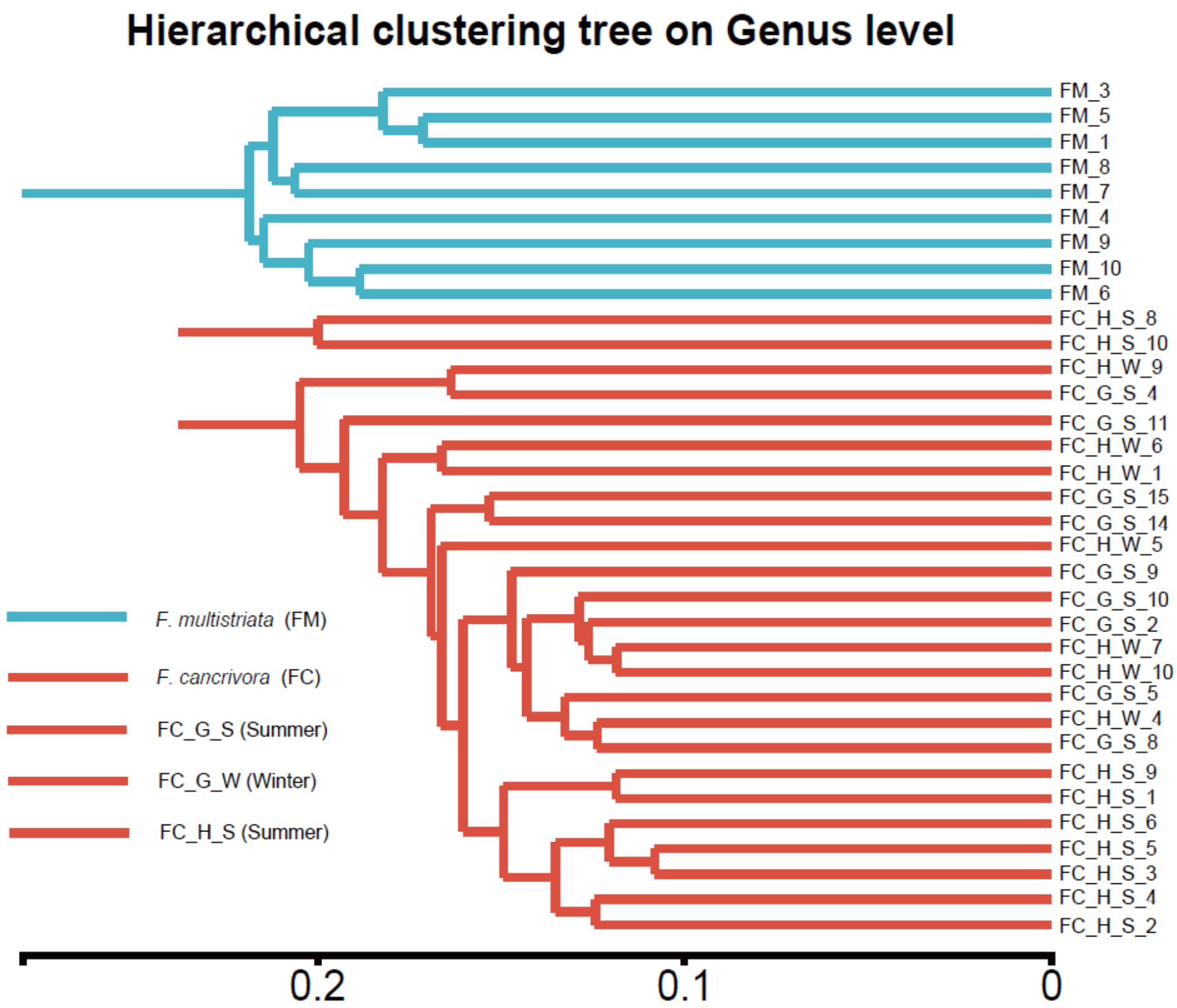

Supplementary Fiugre 3. Gut microbiota clustering: Hierarchical clustering tree at the Genus level, displaying the compositional relationships of gut microbiota among FM and various FC subgroups.

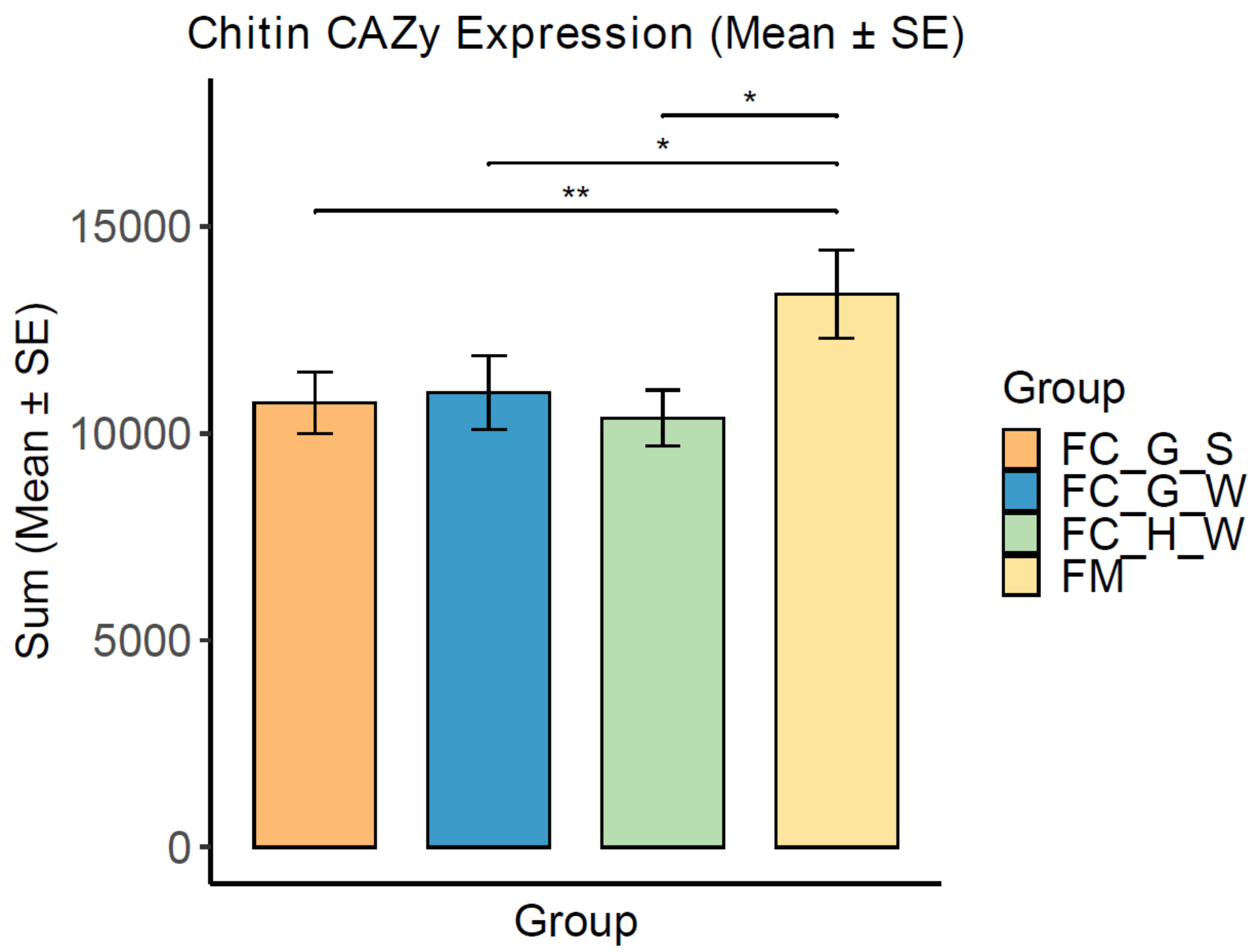

Supplementary Figure 4. Gut microbiome Chitin CAZy expression: Comparison of the sum of gut microbiome chitin CAZy expression across different FC groups (FC_G_S, FC_G_W, FC_H_W) and the FM group. The expression in the FM group is significantly higher than in all FC groups (* : P-value <0.05; **: P-value < 0.01).

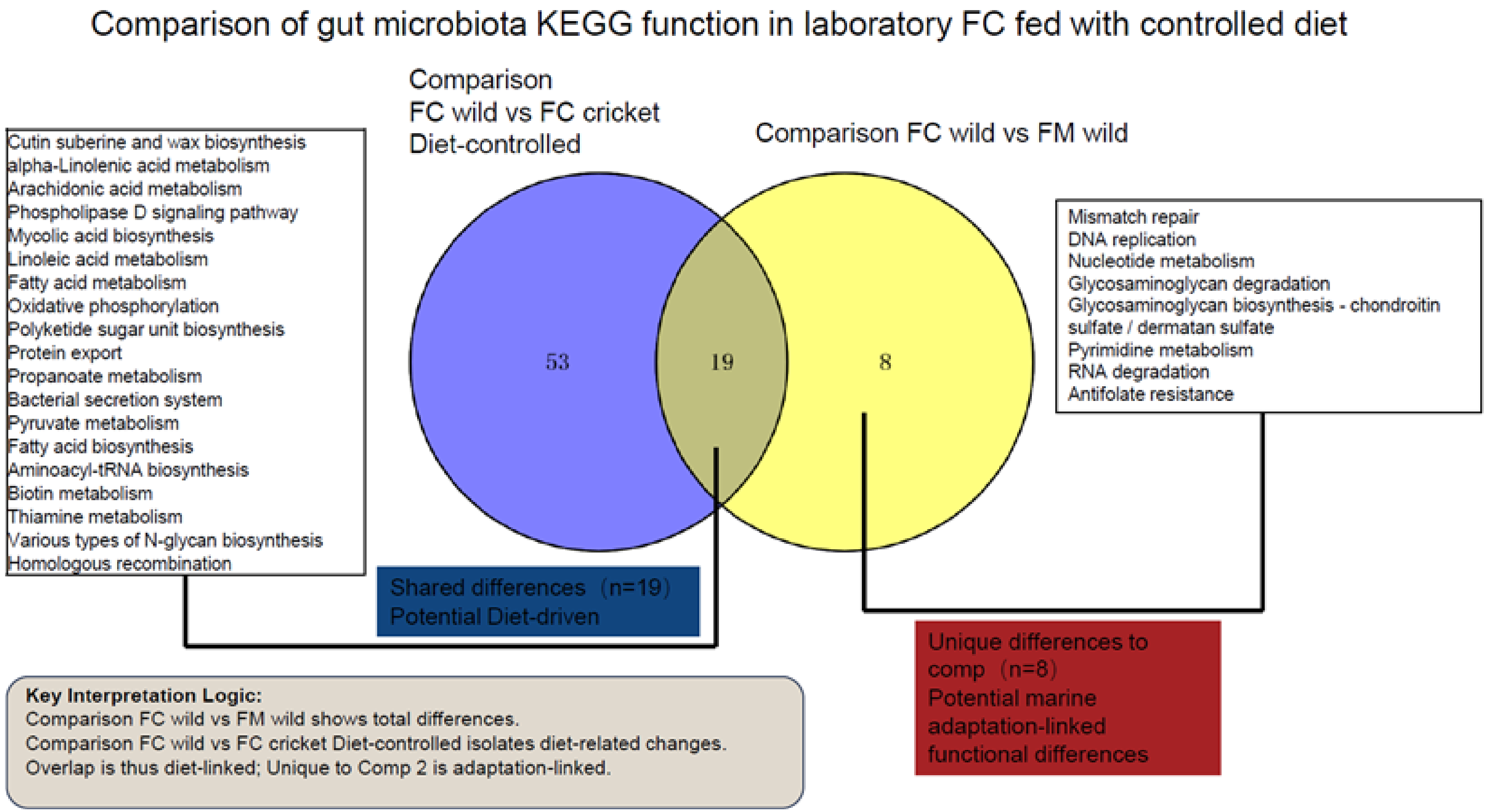

Supplementary Figure 5. KEGG functional analysis of gut microbiota: Venn diagram comparing functional differences between "wild FC vs. diet-controlled FC" and "wild FC vs. wild FM". The overlapping region (n=19) represents potential diet-driven functional differences, while the unique region in the wild FC vs. wild FM

comparison (n=8, e.g., DNA replication, mismatch repair) indicates potential functional differences linked to marine adaptation.
